# An abrupt shift in gross primary productivity over Eastern China-Mongolia and its inter-model diversity in land surface models

**DOI:** 10.1038/s41598-023-49763-1

**Published:** 2023-12-27

**Authors:** Danbi Lee, Jin-Soo Kim, So-Won Park, Jong-Seong Kug

**Affiliations:** 1 Division of Environmental Science and Engineering, Pohang University of Science and Technology (POSTECH), Pohang, South Korea; 2 Low-Carbon and Climate Impact Research Centre, School of Energy and Environment, City University of Hong Kong, Hong Kong, People’s Republic of China

**Keywords:** Biogeochemistry, Climate change

## Abstract

The terrestrial ecosystem in East Asia mainly consists of semi-arid regions that are sensitive to climate change. Therefore, gross primary productivity (GPP) in East Asia could be highly variable and vulnerable to climate change, which can significantly affect the local carbon budget. Here, we examine the spatial and temporal characteristics of GPP variability in East Asia and its relationship with climate factors over the last three decades. We detect an abrupt decrease in GPP over Eastern China-Mongolia region around the year 2000. This is attributed to an abrupt decrease in precipitation associated with the phase shift of the Pacific decadal oscillation (PDO). We also evaluate the reproducibility of offline land surface models to simulate these abrupt changes. Of the twelve models, eight were able to simulate this abrupt response, while the others failed due to the combination of an exaggerated CO_2_ fertilization effect and an underrated climate impact. For accurate prediction, it is necessary to improve the sensitivity of the GPP to changes in CO_2_ concentrations and the climate system.

## Introduction

Land carbon sink is a major driver of the interannual variability of atmospheric CO_2_ concentrations^1–3^. Gross primary productivity (GPP), the organic carbon sequestrated by terrestrial plants through photosynthesis^4^, has a significant impact on the variability of the land carbon sink at regional and global scales^5,6^. GPP is largely modulated by global environmental changes, such as CO_2_ concentrations and meteorological factors^5^. For instance, GPP increases in response to rising atmospheric CO_2_ concentrations, which is referred to as the CO_2_ fertilization effect^7,8^. In addition, the productivity of terrestrial ecosystems significantly depends on climate conditions such as precipitation and temperature^3,9,10^. Thus, the amount of land carbon sequestration varies with these factors and could exhibit strong interannual variability^11^. Moreover, strong meteorological forcing can cause abrupt changes in GPP, which are non-linear and non-stationary^12^. For example, lower absolute soil moisture leads to a prominent abrupt decrease in GPP in arid regions of Europe^13^. Specifically, these abrupt decreases in terrestrial carbon fluxes can lead to a deterioration in local carbon uptake.

Previous studies have reported abrupt changes in vegetation productivity and the underlying mechanisms in semi-arid ecosystems. Ma et al.^14^ have examined how climate extremes impact semi-arid ecosystems in southeastern Australia, with abrupt shifts in phenology and vegetation productivity in drought years. Berdugo et al.^15^ has shown the ubiquity of abrupt productivity loss in global drylands due to climatic, edaphic, and human factors from 2000 to 2019. They show that abrupt decreases in Normalized Difference Vegetation Index (NDVI) have been detected mainly in semi-arid forest regions^15^. As semi-arid ecosystems are highly sensitive to water availability^15,16^, drought or negative trend in precipitation could lead to abrupt decreases in vegetation productivity or loss of ecosystem resilience^14,17^.

East Asia is known to be a significant contributor to the global carbon cycle^7,18,19^. Fossil fuel CO_2_ emissions in East Asia are about 1.5 PgC year^−1^, and about 13–27% of these emissions have been offset by its terrestrial ecosystem from 1990 to 2009^7^. Based on the prevailing semi-arid regions and historical extreme climate events in Inner East Asia^20^, it is possible that there have been abrupt changes in GPP over East Asia^20^. However, there is a limited number of studies on the abrupt changes in GPP, particularly the decrease in this region. Although several studies have detected the change points of GPP in East Asia^4,5,16^, they have mostly focused on greening and monotonic increasing trend of GPP. Furthermore, it is unclear whether current land models can reproduce or simulate the abrupt change in this region.

The primary objective of this study is to identify the major variability and occurrence of abrupt changes in GPP in East Asia over the last three decades. In detail, we examine the dominant spatio-temporal characteristics of GPP variability, detect the significant abrupt changes in GPP, and identify their main drivers. We utilize multiple GPP datasets to validate the consistency of the results. Furthermore, we assess the capabilities of land models to simulate the variability and abrupt changes in GPP.

## Results

### The abrupt shift in GPP over Eastern China-Mongolia

**Figure 1:**
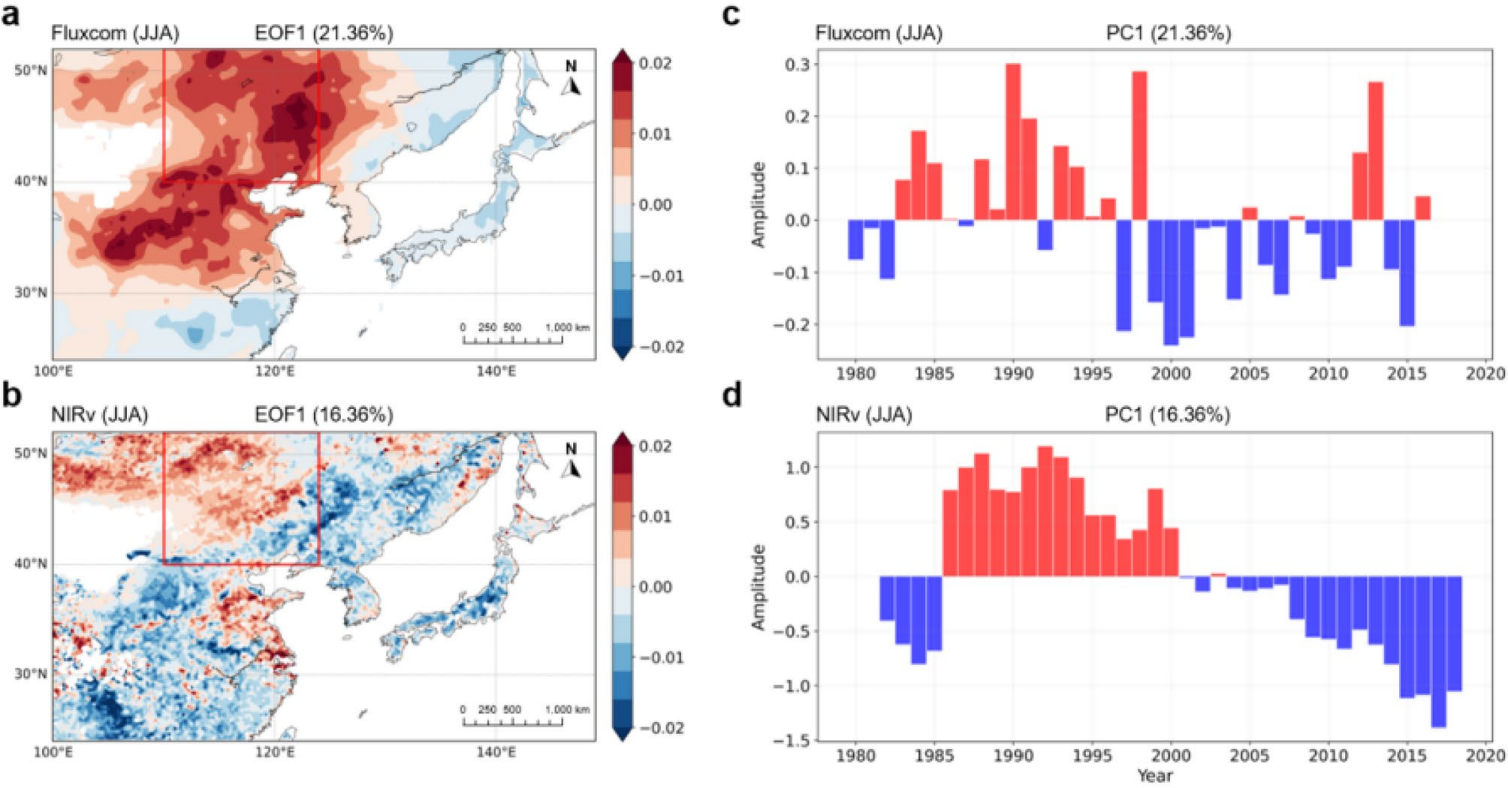
(**a**, **b**) Eigenvectors of the first leading mode and (**c**, **d**) its principal components (PCs) time series from the Empirical Orthogonal Function (EOF) analysis of JJA (June–July–August) GPP_FLUXCOM_ (1980–2016) and GPP_NIRv_ (1982–2018) over East Asia (24°–52°N and 100°–149°E). The values of explained variance for GPP_FLUXCOM_ and GPP_NIRv_ are 21.4% and 16.4% of the total variance, respectively. The red box in a and b indicates Eastern China-Mongolia region (40°–52°N and 110°–124°E).

To investigate the dominant spatio-temporal characteristics of GPP variability, we examine the first leading mode of GPP in East Asia using the Empirical Orthogonal Function (EOF) (see “Data and methods” section, Fig. 1). The first eigenvectors and principal components (PCs) of GPP_FLUXCOM_ and GPP_NIRv_ explain 21.4% and 16.4% of the total variance, respectively. The maximum variation of GPP_FLUXCOM_ and GPP_NIRv_ is consistently located in Eastern China-Mongolia region (40°–52°N and 110°–124°E). Furthermore, the PCs of both first leading modes consistently show a distinctive phase shift around the year 2000. Specifically, there is a positive phase until the 2000s and afterward a negative phase, suggesting a decrease in vegetation productivity around the year 2000 in Eastern China-Mongolia region. In this region, the first eigenvectors and PCs of GPP_FLUXCOM_ and GPP_NIRv_ explain 41.2% and 31.2% of the total variance, respectively (Supplementary Fig. 1). Note that the phase shift in GPP with decadal time scale is comparable in magnitude to their interannual variability, and even larger for GPP_NIRv_ in particular.

**Figure 2:**
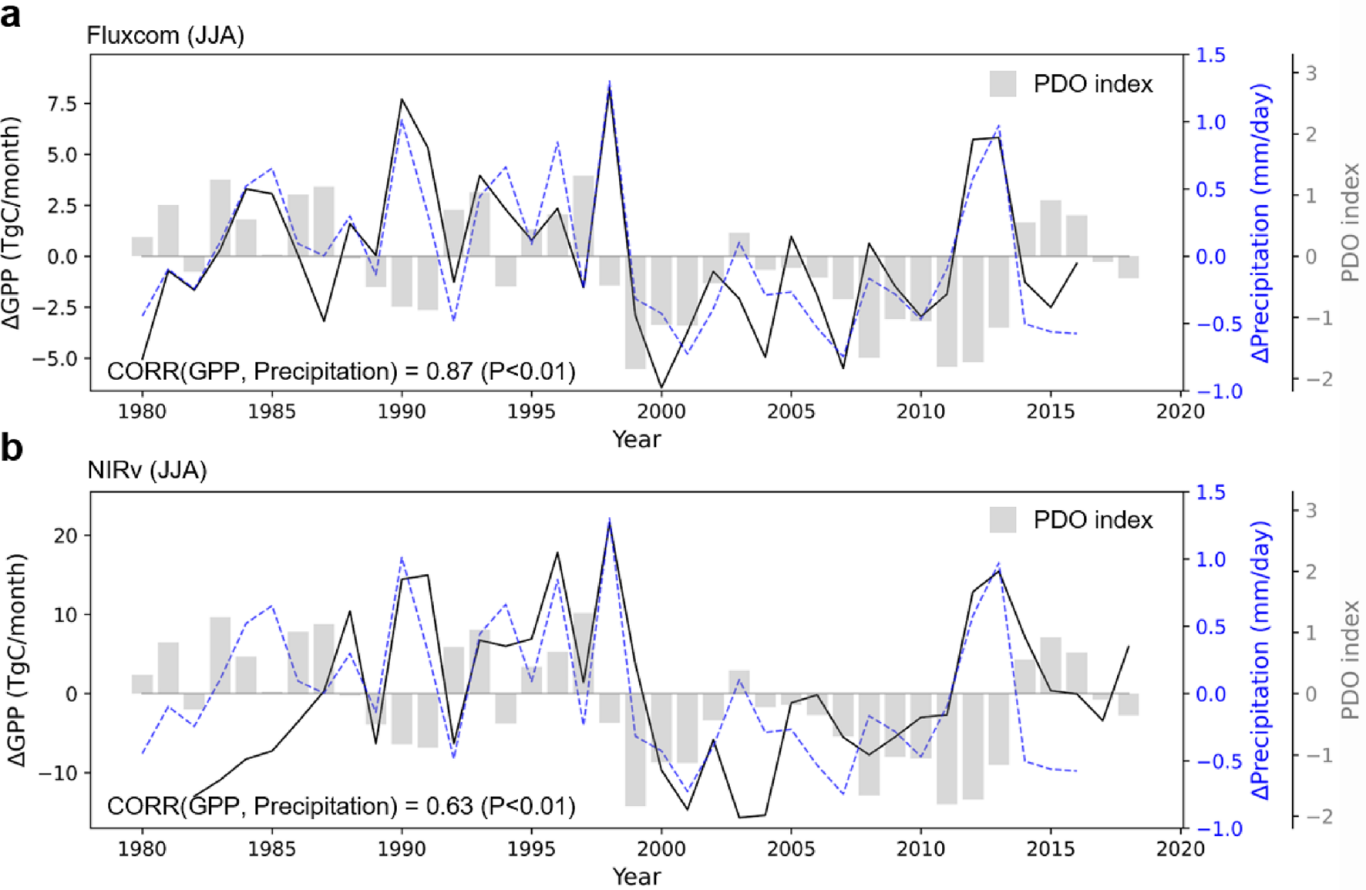
(**a**) Time series of JJA mean GPP_FLUXCOM_ anomaly (1980–2016, black solid line), CRUNCEP precipitation anomaly (1980–2016, blue dashed line), and the Pacific decadal oscillation (PDO) index (1980–2018, gray bar). (**b**) Same as (**a**), but for GPP_NIRv_ anomaly (1982–2018, black solid line). GPP and precipitation anomalies are averaged over Eastern China-Mongolia region (40°–52°N and 110°–124°E) as shown in the red box in Fig. 1.

Eastern China-Mongolia is a semi-arid temperate grassland region with limited precipitation^21,22^. Previous studies have reported that vegetation productivity is strongly dependent on water availability in semi-arid regions across Northern China^16,22^, suggesting the possibility that changes in GPP are caused by changes in precipitation over Eastern China-Mongolia region. Therefore, we further examined the changes in GPP and precipitation in this region and their relationship (Fig. 2). There is a prominent decreasing shift in GPP in the late 1990s; a positive anomaly before the late 1990s and a negative anomaly afterward. This result is consistent with the PCs of the first leading mode shown in Fig. 1c and d. The pronounced decreasing shift in precipitation in the late 1990s is also observed. This is consistent with previous studies that found a decreasing shift in summer precipitation in Northeastern and North China in the late 1990s^23,24^. From 1999 to 2007, the average GPP_NIRv_ (GPP_FLUXCOM_) decreased by 11% (6%) compared to the period of 1990 to 1998, and precipitation also decreased by 28% during the same period. The decreases of GPP_NIRv_ and GPP_FLUXCOM_ in 1998 and 2001 are 36.3 and 12.0 TgC month^−1^, respectively. These changes are about four times larger than the standard deviation of GPP_NIRv_ (1982–2018) and GPP_FLUXCOM_ (1980–2016).

The GPP_FLUXCOM_ and GPP_NIRv_ anomalies are positively correlated with the precipitation anomaly in Eastern China-Mongolia (*r* = 0.87 and 0.63, *P* < 0.01). These findings exhibit similarity across another precipitation dataset (Supplementary Fig. 2). These results are consistent with a previous study that showed a positive relationship between decreasing precipitation and GPP over Northern China from 1999 to 2011^16^. On the contrary, temperature and solar radiation have relatively low correlation coefficients (*r* =  − 0.54, *P* < 0.01 and *r* =  − 0.38, *P* < 0.05) with GPP_FLUXCOM_ anomaly and no statistically significant correlation with GPP_NIRv_ anomaly at the 95% confidence level. These results suggest that the decreasing shift in precipitation is probably responsible for that of GPP in the late 1990s. Moreover, considering the positive correlation between precipitation and evapotranspiration (r = 0.68, P < 0.01) observed in Eastern China-Mongolia, it is conceivable that other processes may contribute to the dramatic shift. For example, changes in the strength of the land–atmosphere coupling can lead to rapid changes in GPP^25^, which will be discussed in more detail in the “Discussion” section.

**Figure 3:**
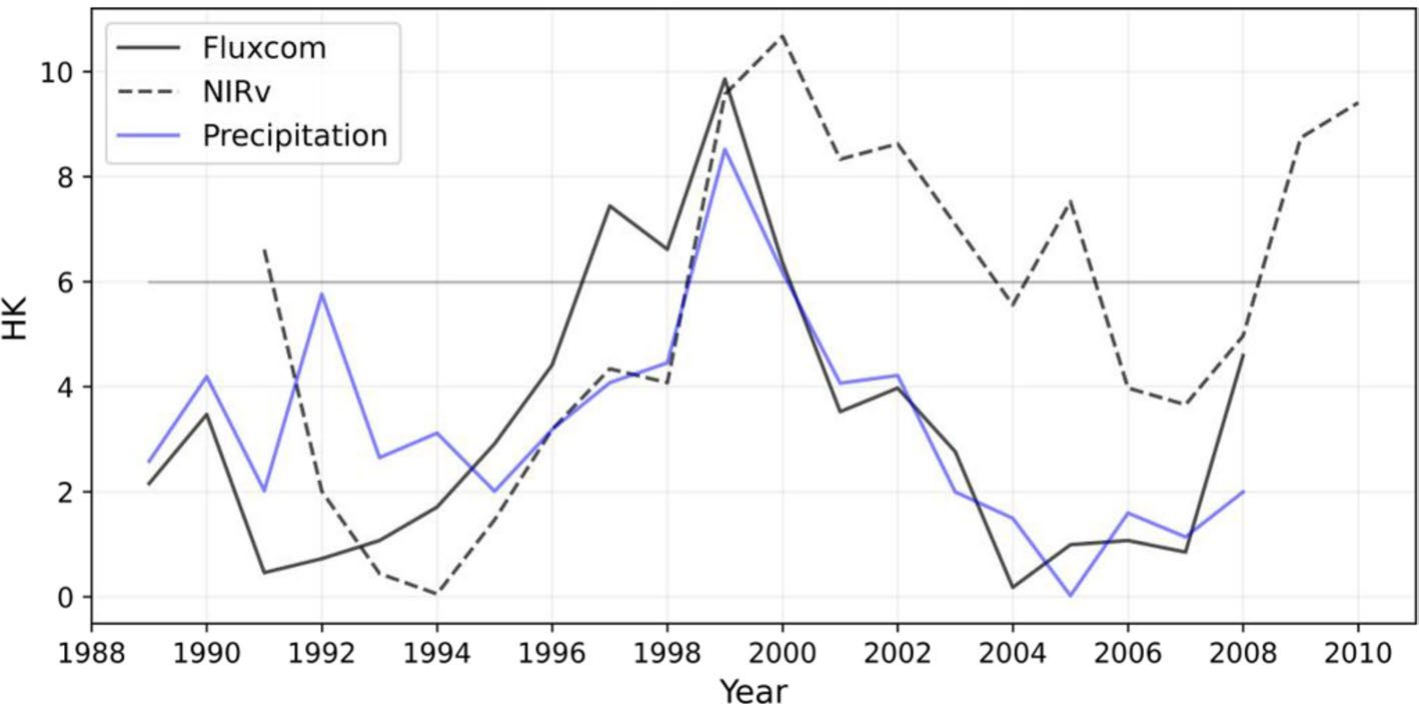
Time series of the Lepage statistic (HK) values of JJA mean GPP_FLUXCOM_ (black solid line), GPP_NIRv_ (black dashed line), and CRUNCEP precipitation (blue solid line) for a window length of 9 years. If the HK value is higher than 5.99, the difference between the means of the two samples is significant at the 95% confidence level. GPP and precipitation anomalies are averaged over Eastern China-Mongolia region (40°–52°N and 110°–124°E) as shown in the red box in Fig. 1.

We further examine whether these decreasing shifts of GPP and precipitation are statistically significant based on the Lepage test (see “Data and methods” section). The phase shifts of GPP_FLUXCOM_, GPP_NIRv_, and precipitation around 1999 are all statistically significant (Fig. 3). GPP_FLUXCOM_ and GPP_NIRv_ show the most significant abrupt shifts in 1999–2000 and 2000–2001, respectively (*P* < 0.05). Precipitation also shows the most significant abrupt shift in 1999–2000 (*P* < 0.05). This result exhibits similarity across another precipitation dataset. (Supplementary Fig. 3). This is consistent with the results of previous studies^23,24^, which showed the abrupt change point of summer precipitation around 1999 over Northeastern China. These highest HK values are largely determined by the standardized Wilcoxon rank sum statistic, indicating that these statistically significant abrupt shifts mostly come from the large changes in the mean state. The results of the Lepage test, conducted with different lengths of the moving window, indicate the consistent abrupt shift year for GPP and precipitation (Supplementary Fig. 4).

**Figure 4:**
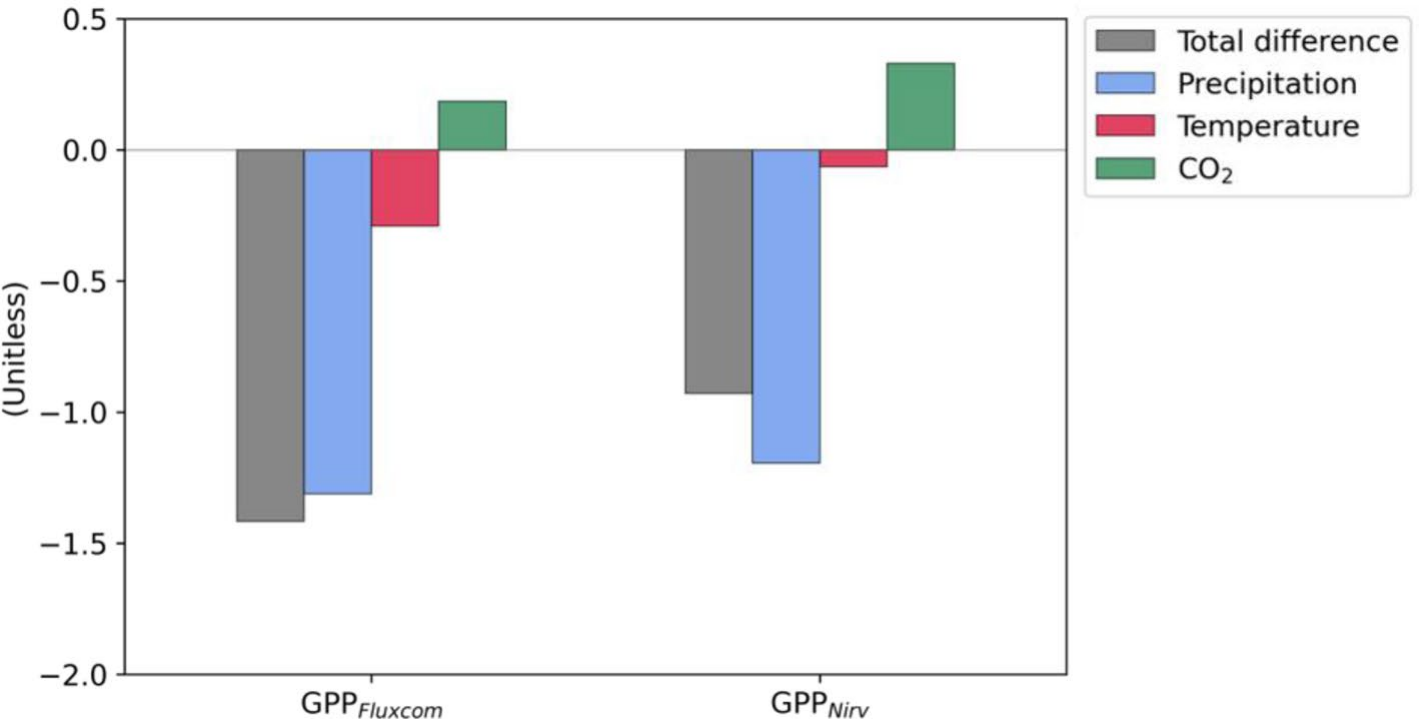
Differences in JJA mean of normalized GPP_FLUXCOM_ and GPP_NIRv_ between P1 (1990 to 1998) and P2 (1999 to 2007), reconstructed from multiple linear regression based on normalized climate factors (precipitation and temperature) and CO_2_. Each bar is the quantitative contribution of precipitation (blue), temperature (red), and CO_2_ (green) to the total difference in GPP (gray). A negative (positive) value indicates a lower (higher) mean state of GPP during P2 compared to P1. All variables are averaged over Eastern China-Mongolia region (40°–52°N and 110°–124°E) as shown in the red box in Fig. 1.

Vegetation productivity is significantly regulated by temperature and CO_2_ concentrations, as well as precipitation^10^. For example, the abrupt changes in NDVI in Southwest China were mainly driven by changes in temperature during 1982–2015^12^. Therefore, to confirm the quantitative contributions of climate factors and CO_2_ to the regime shift in GPP around 1999, we evaluate the relative contributions of changes in temperature, precipitation, and CO_2_ concentrations to the GPP shift using the multiple linear regression method (see “Data and methods” section). With this method, we reconstructed GPP using climate factors and CO_2_, estimating the contributions of the climate and CO_2_ factors to the GPP shift between period 1 (P1: 1990 to 1998) and period 2 (P2: 1999 to 2007). It is evident that there is a decrease in the reconstructed GPP_FLUXCOM_ (− 1.42) and GPP_NIRv_ (− 0.93) between P1 and P2, indicating a significant decrease in GPP during P2 (Fig. 4). It is clear that the decrease in GPP is mostly due to the change in precipitation (GPP_FLUXCOM_: − 1.31, GPP_NIRv_: − 1.19). Temperature also contributes to the decrease, but its impact is minor (GPP_FLUXCOM_: − 0.29, GPP_NIRv_: − 0.064). Increasing CO_2_ leads to an increase in GPP in P2 due to the fertilization effect^7,26^. These results indicate that the change in precipitation is mainly responsible for the statistically significant regime shift in GPP around 1999. In summary, there is a significant abrupt change in GPP in Eastern China-Mongolia over the past three decades, which can be attributed to the abrupt decrease in precipitation.

### The inter-model diversity of the abrupt shift in GPP over Eastern China-Mongolia

**Figure 5:**
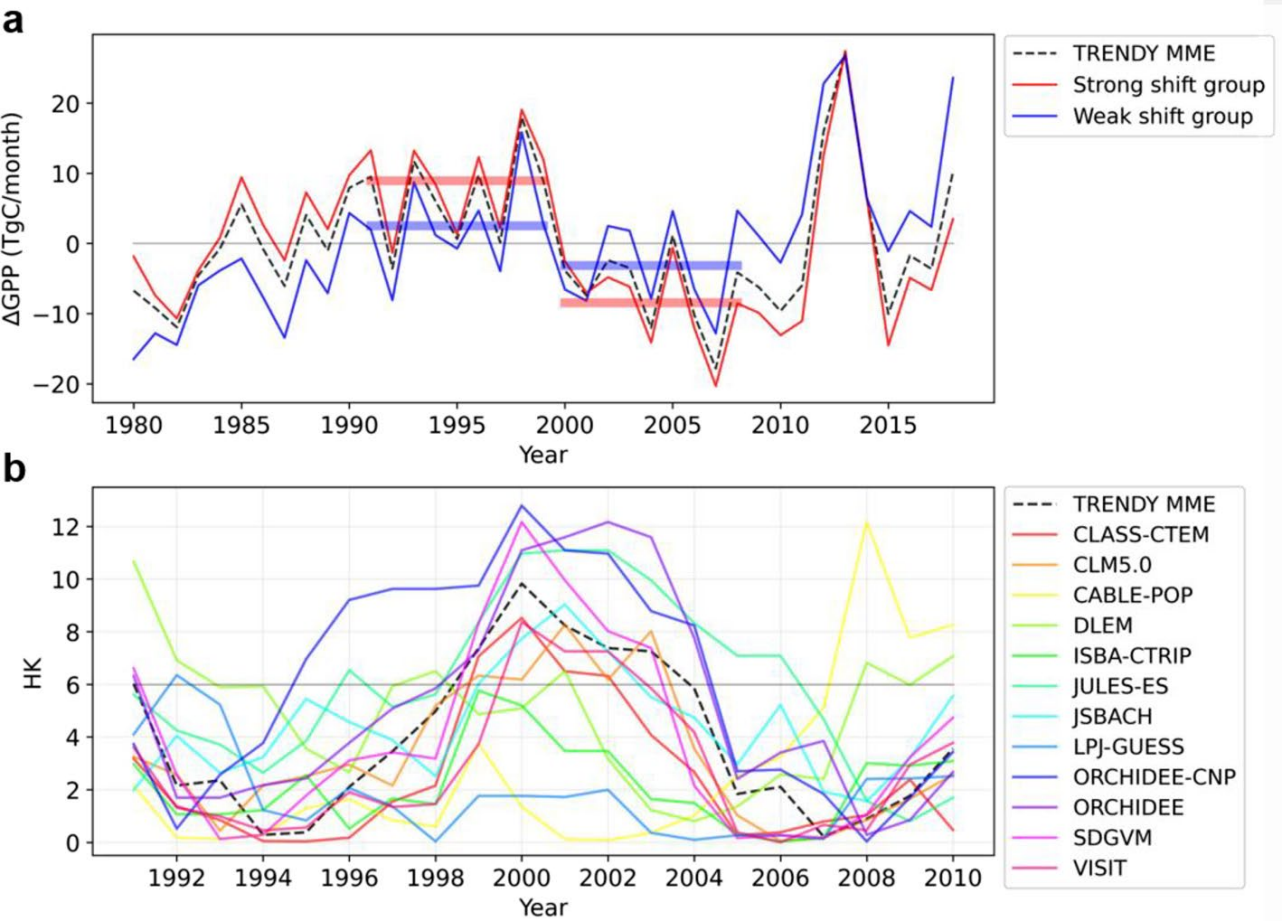
(**a**) Time series of JJA mean GPP anomalies: multi-model ensemble (MME) mean of GPP_TRENDY_ (black dashed line), the mean of the strong shift group (red solid line), and the weak shift group (blue solid line). The red (blue) horizontal bars show the mean values of GPP during P1′ (1991 to 1999) and P2′ (2000 to 2008) of the strong shift group (the weak shift group). (Strong shift group: CLASS-CTEM, CLM5.0, JULES-ES, JSBACH, ORCHIDEE-CNP, ORCHIDEE, SDGVM, and VISIT; Weak shift group: LPJ-GUESS, CABLE-POP, DLEM, and ISBA-CTRIP). (**b**) Time series of Lepage statistics (HK) values of MME mean of JJA GPP_TRENDY_ (black dashed line) and individual TRENDY models (colored solid lines) for a window length of 9 years. If the HK value is higher than 5.99, the difference between the means of the two samples is significant at the 95% confidence level. GPP anomalies are averaged over Eastern China-Mongolia region (40°–52°N and 110°–124°E) as shown in the red box in Fig. 1.

To support the robustness of the regime shift in GPP, we further analyze the output of offline land surface models participating in TRENDY. Using the simulations provided by TRENDY, we isolate the time-varying GPP_climate-forcing_ and the GPP_CO2-forcing_ (see “Data and methods” section). As shown in Fig. 5a, an abrupt shift in GPP around the year 2000 is well captured in the multi-model ensemble (MME) mean of the TRENDY models. This change is statistically significant based on the Lepage test (*P* < 0.05) (Fig. 5b). However, there is significant diversity and spread among the individual TRENDY models, especially in their HK values (Fig. 5b). Of the twelve models, only eight models show a significant abrupt shift in GPP in 2000–2001 (Strong shift group: CLASS-CTEM, CLM5.0, JULES-ES, JSBACH, ORCHIDEE-CNP, ORCHIDEE, SDGVM, and VISIT), while the other models do not simulate abrupt shifts (Weak shift group: LPJ-GUESS, CABLE-POP, DLEM, and ISBA-CTRIP).

We further investigate the differences in GPP between the strong and weak shift groups (Fig. 5a). The red and blue horizontal bars show the mean values of GPP for the two groups during period 1 (P1′: 1991 to 1999) and period 2 (P2′: 2000 to 2008). Although the two groups show quite similar interannual variability, their decadal variability is different. The weak shift group shows a smaller mean state change (− 5.64 TgC month^−1^) in GPP between the two periods than the strong shift group (− 17.4 TgC month^−1^).

**Figure 6:**
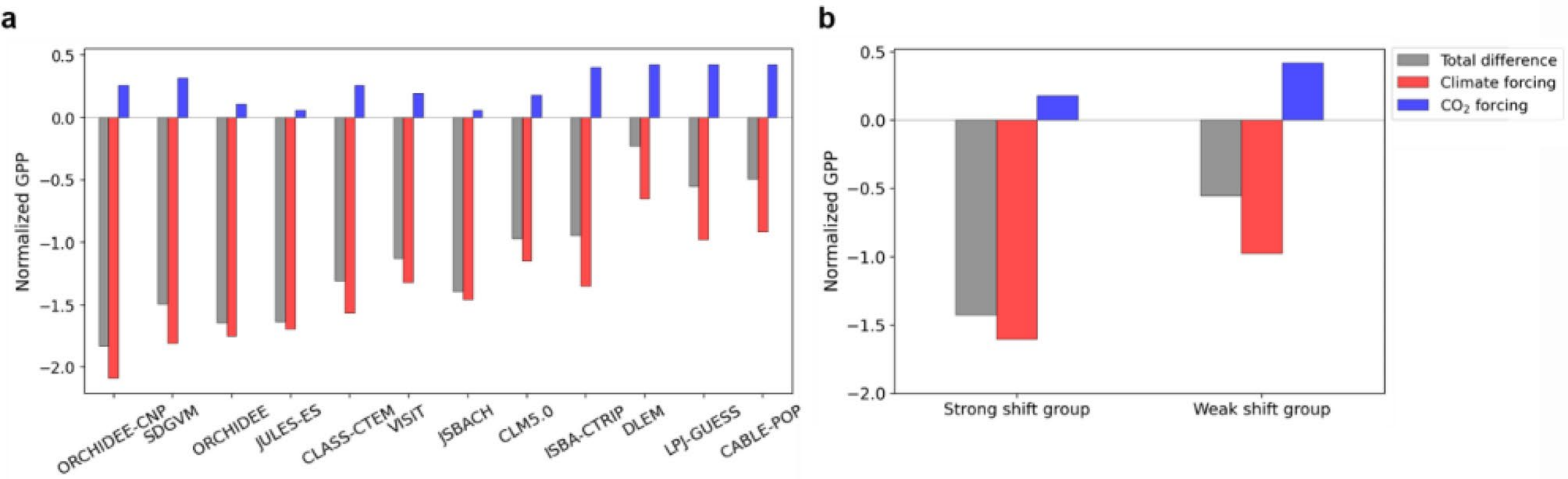
(**a**) Differences in JJA mean of normalized GPP between P1′ and P2′ for the individual TRENDY models. Each bar is the quantitative contribution of climate (red) and CO_2_ forcing (blue) to the total difference in normalized GPP (gray). The models are arranged in order of the magnitude of the HK values. (**b**) Difference in JJA mean of normalized GPP between P1′ and P2′ from the strong and weak shift groups. A negative (positive) value indicates a lower (higher) mean state of GPP during P2′ compared to P1′. GPP anomalies are averaged over Eastern China-Mongolia region (40°–52°N and 110°–124°E) as shown in the red box in Fig. 1.

To examine the driver of the abrupt shift in GPP and the cause of the differences between the groups, we quantify the contributions of the climate and CO_2_ forcings to the mean state change in GPP from the TRENDY models. We calculated the differences in normalized GPP separated by each forcing by subtracting the mean of P1′ from P2′ (Fig. 6). In all models, GPP decreases from P1′ to P2′ and is mostly driven by the GPP_climate-forcing_ (Fig. 6a). The decrease in GPP_climate-forcing_ is mostly driven by changes in precipitation rather than temperature and solar radiation (Supplementary Fig. 5). Therefore, in terms of GPP_climate-forcing_, the TRENDY models also present the aforementioned precipitation-induced regime shift in GPP, which is consistent with satellite and reanalysis data.

The effect of CO_2_ forcing is opposite to that of climate forcing, but the impact is relatively small: the MME mean of GPP_CO2-forcing_ differences (0.26) is smaller in magnitude compared to that of GPP_climate-forcing_ (− 1.40). In particular, the four models in the weak shift group (LPJ-GUESS, CABLE-POP, DLEM, and ISBA-CTRIP) have a higher sensitivity of GPP to CO_2_ and a lower sensitivity to climate compared to the other group (Fig. 6a). These characteristics are more obvious in the group means (Fig. 6b): the MME means of the GPP_CO2-forcing_ and GPP_climate-forcing_ differences for the weak shift group (the strong shift group) are 0.42 (0.18) and − 0.98 (− 1.60), respectively. Therefore, the differences in the sensitivity of GPP to CO_2_ and climate forcing between the models lead to significant differences in the changes in GPP between the two groups.

## Discussion

We have investigated the first EOF mode of GPP variability and found significant abrupt changes in GPP in East Asia over the past three decades. The PCs of the first leading modes of GPP_FLUXCOM_ and GPP_NIRv_ consistently show a distinctive phase shift around the year 2000 in Eastern China-Mongolia. The abrupt change in GPP around the year 2000 is consistently detected by satellite, reanalysis datasets, and land surface models. The high correlation between GPP and precipitation, and the abrupt decrease in precipitation indicate that the abrupt decrease in GPP resulted from the changes in precipitation. Quantitative analysis using the land surface models shows that the shift in GPP is mostly driven by changes in precipitation, supporting the GPP-precipitation mechanism.

It has been reported that the Pacific decadal oscillation (PDO) phase change can affect the regional precipitation in East Asia through atmospheric teleconnections^24,28–32^. After the late 1990s, the Pacific entered a negative phase of the PDO (Fig. 2), and the upper-level atmospheric circulation changed accordingly. The East Asian westerly jet stream (EAWJS) is weakened and poleward shifted. The poleward-shifted EAWJS changes the jet-related secondary meridional-vertical circulation^29^, making an anomalous descending motion to the northern parts of East Asia. Subsequently, the anticyclonic circulation dominates the target region in a negative PDO phase, resulting in less precipitation in the late 1990s. Therefore, the precipitation shifts around the year 2000 are related to the phase shift of the PDO. Our results suggest that the abrupt changes in GPP in Eastern China-Mongolia region depend on the phase change of the PDO.

Of the twelve offline land surface models, eight were able to simulate this abrupt response, while the others failed to capture it. The differences between the TRENDY models can be attributed to the diverse sensitivities of CO_2_ and climate forcing. This implies that differences in the sensitivity of GPP to CO_2_ and climate forcing between the models resulted in the inter-model diversity in simulating the abrupt changes. One factor contributing to the differences in sensitivity is that terrestrial photosynthetic CO_2_ assimilation, which is the basis for model estimation of GPP, is represented and parameterized differently across terrestrial biosphere models^33^. This leads to different responses of GPP to key environmental variables, including climate and CO_2_ forcing^33^. Therefore, this leads to structural differences (e.g., sensitivities) and large uncertainties in the models^34^. Therefore, improving the response and parameterization of photosynthesis in each model would be helpful to reduce the uncertainties in the TRENDY models and investigate the more accurate variability of GPP in Eastern China-Mongolia.

It is possible that land–atmosphere coupling, a positive feedback between vegetation and precipitation recycling^25^, is involved in the abrupt change in GPP over Eastern China-Mongolia. In this feedback loop, reduced precipitation in water-limited areas leads to a decline in vegetation, which can decrease evapotranspiration. A decrease in precipitation due to the reduced evapotranspiration is expected to lead to a decrease in vegetation. The Eastern China-Mongolia region is known to have a high precipitation recycling ratio^35^; in other words, precipitation is highly contributed by evapotranspiration from the same region. Thus, precipitation and evapotranspiration have a positive correlation (r = 0.68, *P* < 0.01) from 1990 to 2007. Accordingly, evapotranspiration also shows a significant abrupt shift in 1999–2000 in the Eastern China-Mongolia region (*P* < 0.05). Given these, it is conceivable that the positive feedback could contribute to the dramatic shift in GPP and its persistence. Due to the significant effects of the feedback, further studies are needed to understand the role of land–atmosphere coupling in abrupt changes in vegetation productivity.

Semiarid carbon flux is known to be influenced not only by precipitation, but also by temperature, as warming-induced water deficit also leads to a decrease in photosynthesis^1^. In this study, compared to precipitation, temperature exhibits a relatively low correlation coefficient (r =  − 0.54, *P* < 0.01) with GPP_FLUXCOM_ anomaly in 1980–2016 and no statistically significant correlation with GPP_NIRv_ anomaly at the 95% confidence level. However, over a shorter time period (1998–2016), GPP_FLUXCOM_ and GPP_NIRv_ anomalies have a stronger negative correlation with temperature anomaly (r =  − 0.68, *P* < 0.01 and − 0.56, *P* < 0.05). However, this relationship was not considered in this study. Indeed, GPP showed a delayed response to the recovery of precipitation in 2003–2004 (Fig. 2). Based on the negative correlation between GPP and temperature, it can be suggested that the positive temperature anomaly in the early 2000s may have contributed to the delay in GPP recovery in 2003–2004 (figure not shown). Therefore, it is necessary to consider temperature to understand the overall variability of GPP in East Asia comprehensively.

The GPP time span (Fluxcom, 1950–2016; NIRv, 1982–2018; Trendy, 1700–2018) does not account for recent changes in GPP. A recent abrupt shift towards a hotter and drier climate in inner East Asia^20^ could indicate the possibility of frequent abrupt changes in GPP. Thus, it is necessary to conduct an extended study to investigate the impacts of a hotter and drier climate on GPP in inner East Asia. In addition, future abrupt changes in vegetation productivity in East Asia need to be investigated. Drought risks in East Asia (30°–55°N and 110°–140°E) would increase due to rapidly rising evaporative demand^27^. Moreover, the projections based on the representative concentration pathways (RCPs) RCP8.5 and RCP4.5 indicate that East Asia (30°–70°N and 80°–180°E) is one of the regions that will experience the most significant expansion of drylands by the end of the twenty-first century^36,37^. The high correlation between water availability and GPP suggests that abrupt changes in vegetation productivity may become more widespread and frequent in East Asia in the future. Furthermore, the drylands of East Asia will play an important role in regulating the interannual variability of global dryland GPP^37^. This suggests that the intense occurrence of abrupt changes could exacerbate the interannual variation of the global land sink in the future. In this regard, it is essential to conduct further research on future abrupt changes in vegetation productivity and terrestrial carbon flux. Further research should focus on the northeastern part of East Asia, where dryland expansion is expected^27^, and the western part of East Asia, where future drought risks are expected to be higher^37^. This further research could contribute to developing carbon–neutral strategies by enhancing the understanding of the interannual variability of the land sink.

## Data and methods

### GPP datasets

Terrestrial photosynthesis is a highly uncertain process due to the lack of direct observations of GPP on a global scale. To obtain robust results on GPP responses to climate variability, we used both data-driven and process-based GPP datasets with over 30 years of records for the East Asian region.

### FLUXCOM

FLUXCOM (version RS + METEO) provides ensembles of upscaled GPP products on a global scale (www.fluxcom.org) for the period from 1950 to 2016^38,39^. FLUXCOM GPP was developed using machine learning techniques to merge FLUXNET site-level observations, satellite remote sensing, and meteorological data to scale these fluxes to the global scale^34,40,41^. This study used the FLUXCOM GPP, which is calculated by averaging GPP ensembles generated from three machine learning algorithms (Random Forest, Artificial Neural Network, and Multivariate Adaptive Regression Splines) and two flux partitioning methods (daytime and nighttime) for the period from 1980 to 2016^42^.

### Near-infrared reflectance (NIRv)

Previous studies have shown that both NIRv and SIF effectively capture global-scale changes in terrestrial GPP^43–45^. This study used NIRv due to its broader data coverage and higher resolution compared to satellite-based SIF^46^ to investigate long-term trends in GPP. The NIRv data are derived from the long-term satellite datasets of the Advanced Very High Resolution Radiometer (AVHRR) reflectance from the Land Long Term Data Record v4 (LTDR) product for the period from 1982 to 2018. This study utilizes the dataset for all available time periods. NIRv is calculated as a function of monthly NDVI and near-infrared reflection of the total pixel (NIRT)^43,44^. A subtraction of 0.08 from the NDVI is applied to eliminate the influence of bare soils^46^ [Eq. (1)].

1 $$NIRv=(NDVI-0.08)\times NIRT$$

### TRENDY v8

The model intercomparison project TRENDY v8 provides GPP output from Dynamic Global Vegetation Models (DGVMs) for the period from 1700 to 2018^47^. These models are forced with the same meteorological conditions, atmospheric CO_2_ concentrations, and land use datasets^34,46,48^. TRENDY v8 provides a suite of simulations that allow the response of land to climate, CO_2_, and land use forcing to be separated. The “S0” simulation is a baseline simulation with time-invariant pre-industrial CO_2_ concentrations, climate, and land use. The “S1” simulation is forced by time-varying atmospheric CO_2_ concentrations, but with fixed climate and land use information. The “S2” simulation is forced by time-varying atmospheric CO_2_ concentrations and climate with constant land use change.

This study used the GPP output from 12 DGVMs from 1980 to 2018, which was regridded to a spatial resolution of 1° × 1° for consistent analysis in the multi-model domain (Table 1). Based on the experimental designs, we utilize the experimental simulations to evaluate the relative contributions of CO_2_ fertilization and climate forcing to the change in GPP. For each TRENDY model, the CO_2_ fertilization effect-driven change in GPP (GPP_CO2-forcing_) is calculated from the difference between S1 and S0, while the climate change-driven change in GPP (GPP_climate-forcing_) is calculated from the difference between S2 and S1. In addition, the response of GPP to total forcing (GPP_TRENDY_) is calculated as the difference between S2 and S0, which is the sum of CO_2_ fertilization and climate change-driven GPP. Note that this study excludes the effect of land use change on GPP, as land use change does not appear to be a significant driver of GPP change over the study period (Supplementary Fig. 6).

**Table 1:** Summary of the TRENDY v8 models used in this study.

Model	Spatial resolution	References
CABLE-POP	$1^\circ \times 1^\circ$	Haverd et al.^65^
CLASS-CTEM	$2.8125^\circ \times 2.8125^\circ$	Melton and Arora^66^
CLM5.0	$1.875^\circ \times 0.625^\circ$	Lawrence et al.^67^
DLEM	$0.5^\circ \times 0.5^\circ$	Tian et al.^68^
ISBA-CTRIP	$1^\circ \times 1.2^\circ$	Decharme et al.^69^
JSBACH	$1.875^\circ \times 1.875^\circ$	Mauritsen et al.^64^
JULES-ES	$1.875^\circ \times 1.25^\circ$	Sellar et al.^70^
LPJ-GUESS	$0.5^\circ \times 0.5^\circ$	Smith et al.^71^
ORCHIDEE	$0.5^\circ \times 0.5^\circ$	Krinner et al.^72^
ORCHIDEE-CNP	$2^\circ \times 2^\circ$	Goll et al.^73^
SDGVM	$0.5^\circ \times 0.5^\circ$	Walker et al.^74^
VISIT	$0.5^\circ \times 0.5^\circ$	Kato et al.^75^

We focused on averaged GPP in the boreal summer months of June–July–August (JJA) to examine spatio-temporal variations across the East Asia region (24°–52°N and 100°–149°E). The JJA means of GPP_FLUXCOM_ and GPP_NIRv_ have the highest values and variability, accounting for 78.3% and 78.7% of annual vegetation productivity, respectively (Supplementary Fig. 7).

### Meteorological data

We used precipitation, temperature, and solar radiation data from CRUNCEPv7 for the period 1980 to 2016^49^, along with evapotranspiration data from ERA5 for the period 1980 to 2018^51^. We also utilized precipitation data from GPCC for the period 1982 to 2018 to verify the consistency of the results^52^. Atmospheric CO_2_ concentration data from the NOAA GML Carbon Cycle Cooperative Global Air Sampling Network were also used for the period 1980 to 2016^50^. Table 2 summarizes the meteorological data used in this study.

**Table. 2:** Summary of the meteorological data used in this study.

Dataset	Variables	Data period	Study period
CRUNCEPv7^49^	Precipitation Temperature Solar radiation	1980–2016	1980–2016
ERA5^51^	Evapotranspiration	1979–2021	1980–2018
GPCC^52^	Precipitation	1982–2021	1982–2018
NOAA GML carbon cycle cooperative global air sampling network^50^	Atmospheric CO_2_ concentration	1968–2020	1980–2016

### EOF-based detection of dominant spatial and temporal variability

The EOF analysis is considered to be an effective approach to investigate the temporal and spatial coherence for each orthogonal component of complex time-varying spatial patterns^53^. It performs a linear transformation, defined in terms of the eigenvectors of their covariance matrix, which provides dimension reduction^54^. This analysis allows us to examine the structural features of a long-time series variable and extract its representation of variability. Therefore, it effectively captures representations of climate variability and identifies the impacts of large-scale climate change and abnormal climate on ecosystems^55^. Thus, the EOF analysis has been used to characterize the dominant modes of variability in GPP and vegetation indexes in previous studies^53,56,57^.

### Lepage test for abrupt change detection

There are several methods for detecting change points in adjacent data on a decadal scale. Among these methods, the Lepage test^58^ has few underlying assumptions, which makes it adopted for detecting various types of climate changes, including linear trends, cyclical variations, step-like changes, and discontinuous changes^23^. This method has been used extensively in numerous studies to detect abrupt changes^23,24,59,60^. The Lepage statistic (HK) is calculated as the sum of the squares of the standardized Wilcoxon (W) and Ansari-Bradley (A) statistics [Eq. (2)]^61^, which represent the comparison of the mean and variance between two samples, respectively^58^.

2 $$HK= \frac{{[W-E(W)]}^{2}}{V(W)}+ \frac{{[A-E(A)]}^{2}}{V(A)}$$

where E(W), E(A), V(W), and V(A) stand for the expected value and variance of W and A, respectively. If the HK is greater than 5.99, the difference between the two samples is significant at the 95% confidence level. A detailed description of the methods can be found in an earlier study^23^. In this study, the Lepage test with a 9-year moving window is used to identify the inter-decadal abrupt change point. We defined the year with the largest HK value above 5.99 as the year in which an abrupt change occurred, based on previous studies^24,62^.

### Quantifying the contribution of climate and CO_2_ to the change in GPP

The multiple linear regression method was used to analyze the linear relationship between multiple variables^63^. This method was used to investigate the relative contributions of climatic (temperature and precipitation) and CO_2_ factors to vegetation productivity [Eq. (3)].

3 $$GPP=\alpha Prec +\beta Temp+\gamma {CO}_{2}+\delta$$

where Prec, Temp, and CO_2_ stand for precipitation, temperature, and CO_2_ concentration, respectively. $\alpha$, $\upbeta$, and $\gamma$ represent the partial regression coefficients for each variable. $\delta$ represents the residuals. We used normalized GPP, precipitation, temperature, and CO_2_ concentrations.

### Supplementary Information

Supplementary Information.

## Data Availability

All data used in this study are publicly available and can be downloaded from the corresponding websites (FLUXCOM: https://fluxcom.org/; NIRv: https://data.tpdc.ac.cn/; Access to TRENDY v.8 data can be obtained by contacting Stephen Sitch: (S.A.Sitch@exeter.ac.uk); CRUNCEPv7 datasets (precipitation, temperature, and solar radiation): https://rda.ucar.edu/datasets/ds314.3/citation/; ERA5 datasets (evapotranspiration): https://cds.climate.copernicus.eu/; GPCC (precipitation): https://psl.noaa.gov/; CO_2_ concentration: https://gml.noaa.gov/).

## Code availability

The computer codes that support the analysis within this paper are available from the corresponding author on request.
