# Supplementary material for: An abrupt shift in gross primary productivity over Eastern China-Mongolia and its inter-model diversity in land surface models
Source: Sci Rep. 2023 Dec 27;13:22971. doi: 10.1038/s41598-023-49763-1 (PMC10752903; doi:10.1038/s41598-023-49763-1)
Supplement: Supplementary file 1 — Supplementary Information. [file 41598_2023_49763_MOESM1_ESM.docx]

Supplementary Information for

**An abrupt shift in gross primary productivity over Eastern China-Mongolia and its inter-model diversity in land surface models**

**Danbi Lee^1^, Jin-Soo Kim^2^, So-Won Park*^1^, and Jong-Seong Kug*^1^**

^1^Division of Environmental Science and Engineering, Pohang University of Science and Technology (POSTECH), Pohang, South Korea.
^2^Low-Carbon and Climate Impact Research Centre, School of Energy and Environment, City University of Hong Kong, Tat Chee Avenue, Kowloon, Hong Kong, China.

Corresponding author: So-Won Park (email: sowon@postech.ac.kr),
Jong-Seong Kug (email: jskug@postech.ac.kr)

**
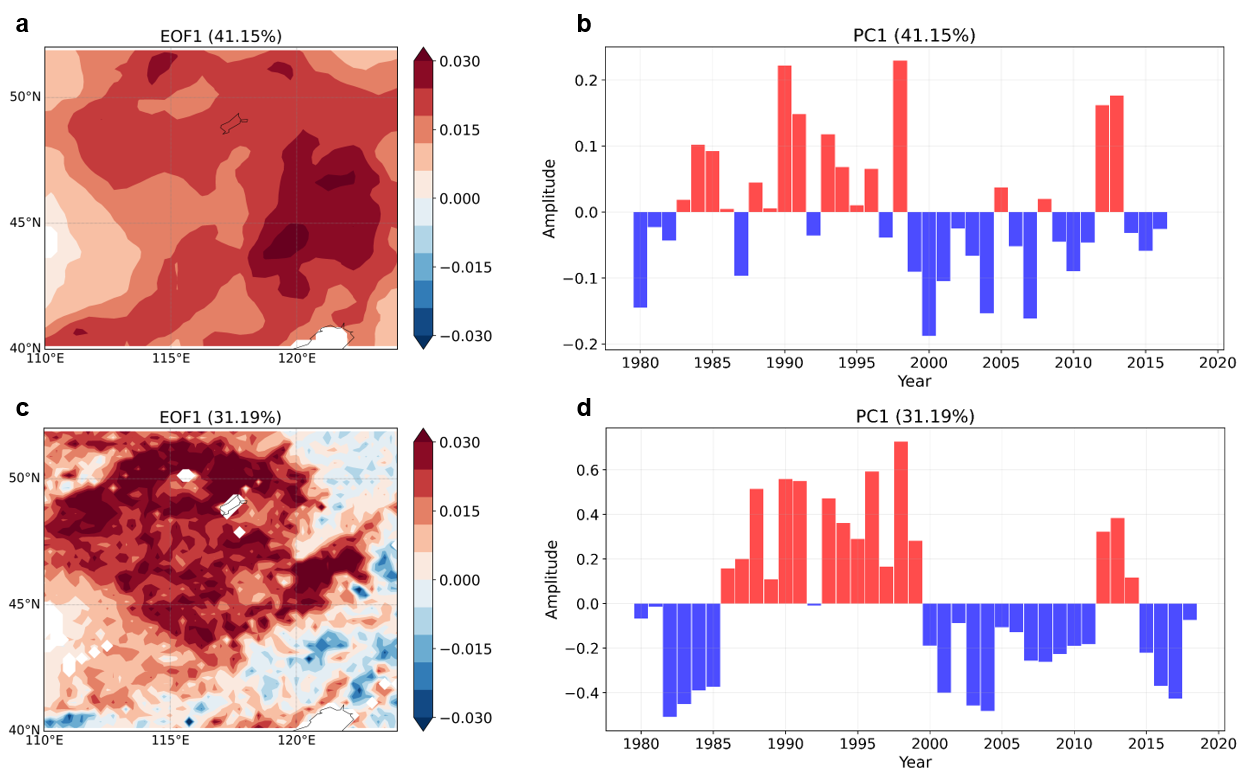
Supplementary Figure. 1** (a, b) Eigenvector of the first leading mode and (c, d) its principal components (PCs) time series from the empirical orthogonal function (EOF) analysis of JJA (June–July–August) GPP_FLUXCOM_ (1980−2016) and GPP_NIRv_ (1982−2018) over Eastern China-Mongolia region (40°–52°N and 110°–124°E). The values of explained variance for GPP_FLUXCOM_ and GPP_NIRv_ are 41.2% and 31.2% of the total variance, respectively.

**
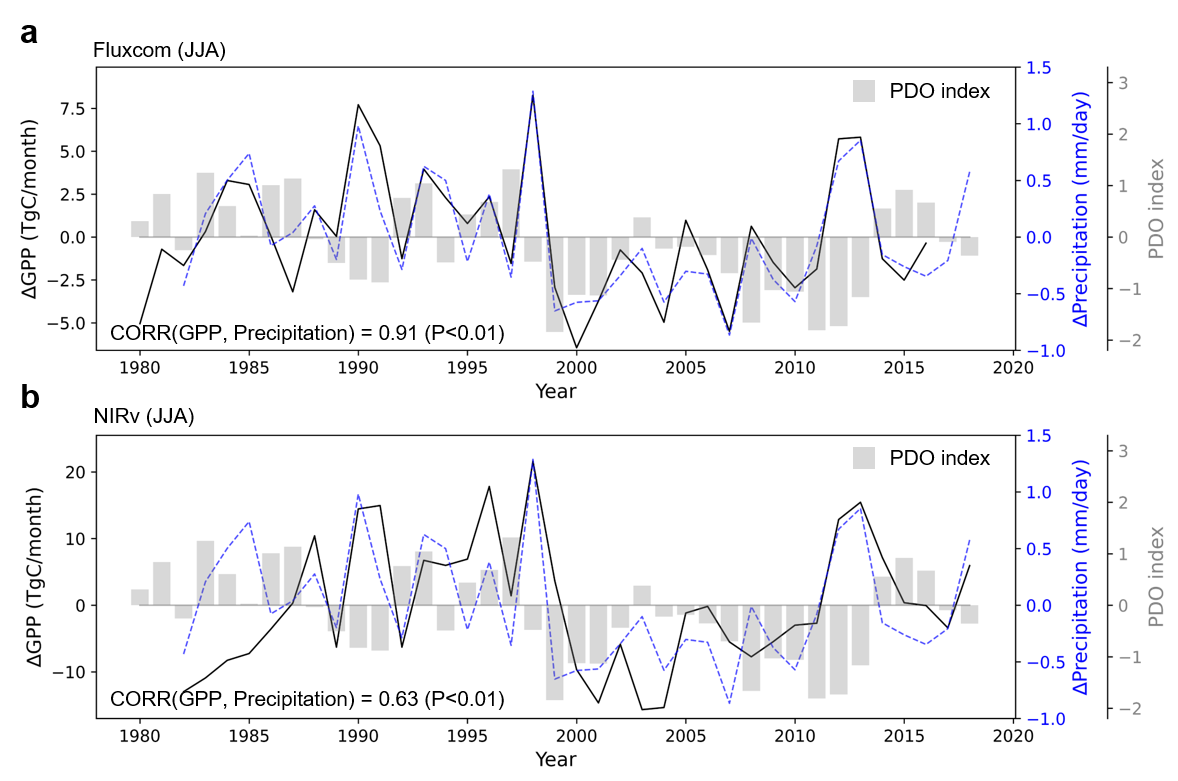
Supplementary Figure. 2** (a) Time series of JJA mean GPP_FLUXCOM_ anomaly (1980−2016, black solid line), GPCC precipitation anomaly (1980−2018, blue dashed line), and the Pacific decadal oscillation (PDO) index (1980−2018, grey bar). (b) Same as (a), but for GPP_NIRv_ anomaly (1982−2018, black solid line). GPP and precipitation anomalies are averaged over Eastern China-Mongolia region (40°–52°N and 110°–124°E) as shown in the red box in Fig. 1.

**
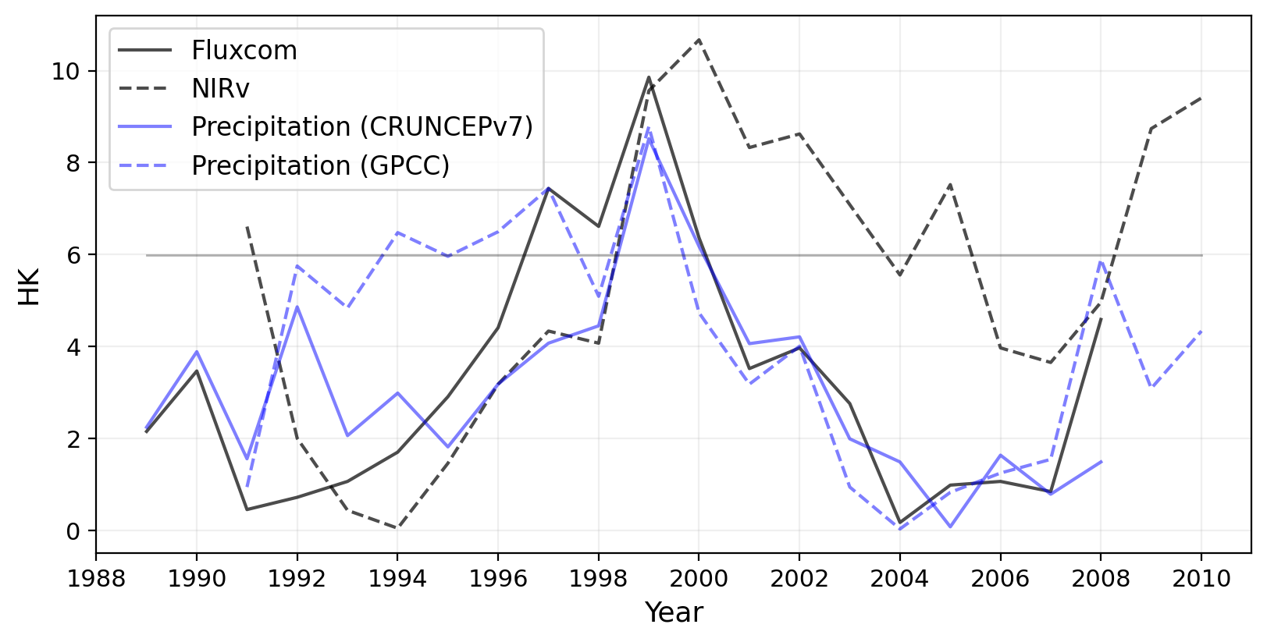
Supplementary Figure. 3** Time series of the Lepage statistic (HK) values of JJA mean GPP_FLUXCOM_ (black solid line), GPP_NIRv_ (black dashed line), CRUNCEP precipitation (blue solid line), and GPCC precipitation (blue dashed line) for a window length of 9 years. If the HK value is higher than 5.99, the difference between the means of the two samples is significant at the 95% confidence level. GPP and precipitation anomalies are averaged over Eastern China-Mongolia region (40°–52°N and 110°–124°E) as shown in the red box in Fig. 1.

**
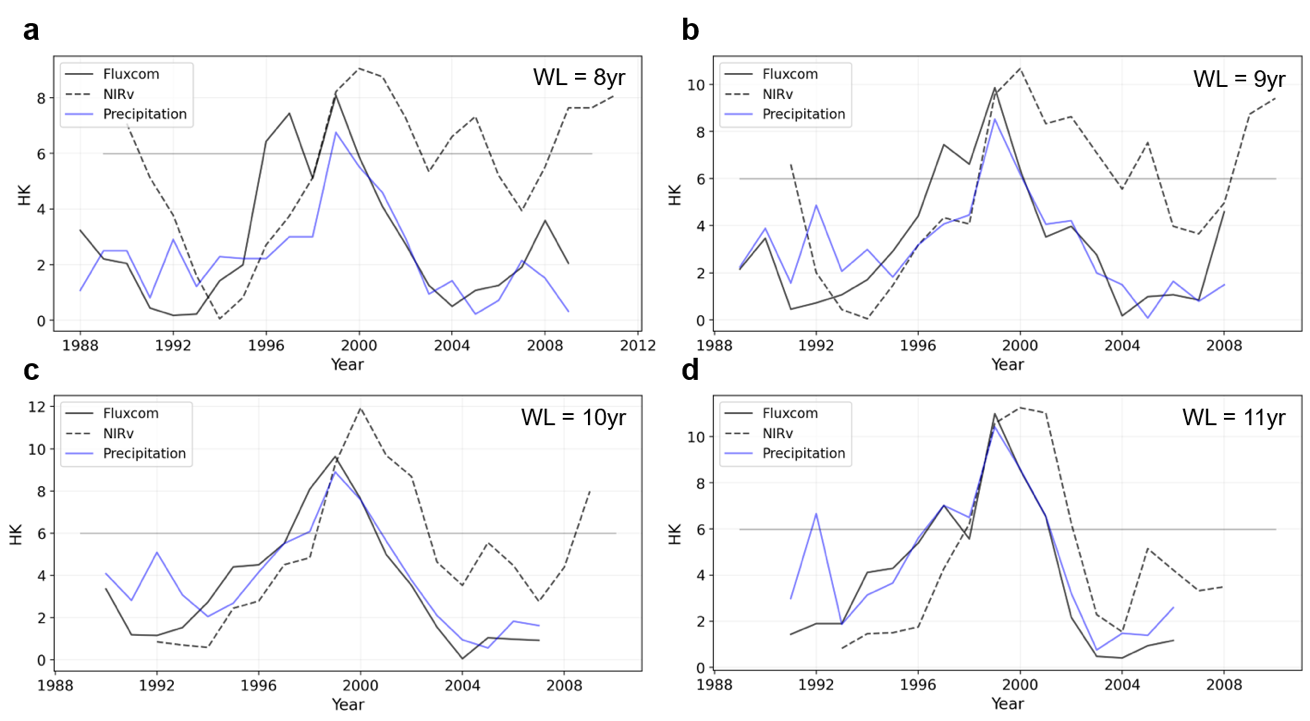
Supplementary Figure. 4** Time series of the Lepage statistic (HK) values of JJA mean GPP_FLUXCOM_ (black solid line), GPP_NIRv_ (black dashed line), and CRUNCEP precipitation (blue solid line). Same as Fig. 3, but for different window lengths (WL).

**
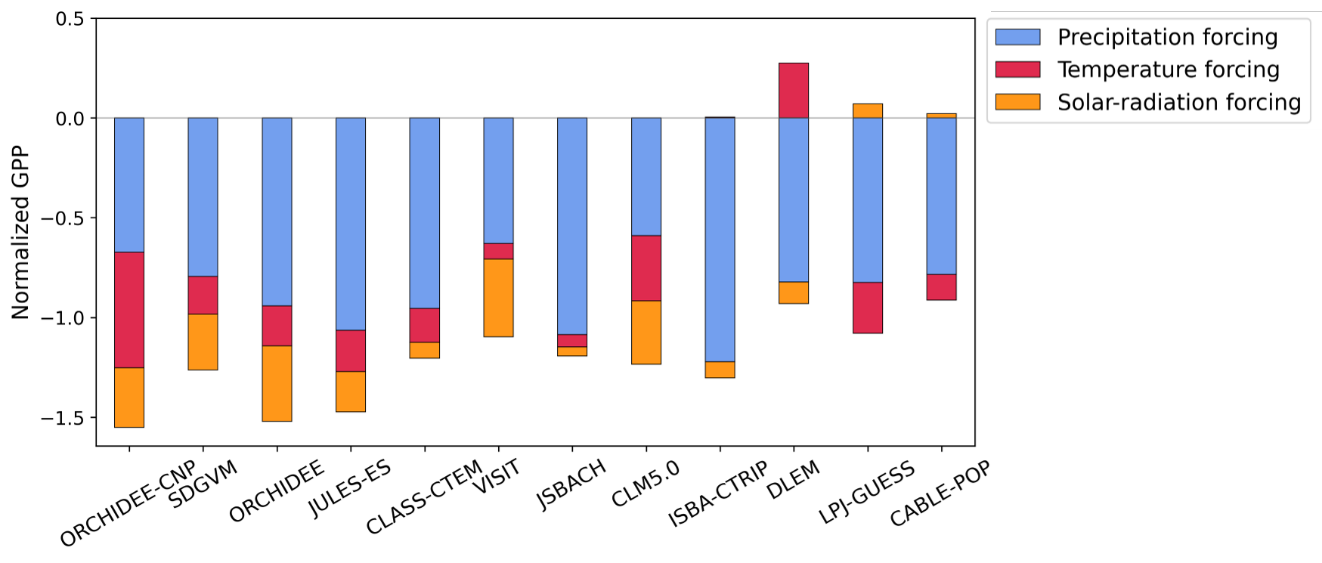
Supplementary Figure. 5** Differences in JJA mean of normalized GPP_climate-forcing_ between P1' (1991 to 1999) and P2' (2000 to 2008) for the individual TRENDY models, reconstructed from multiple linear regression based on normalized climate factors (precipitation, temperature, and solar radiation). Each bar is the quantitative contribution of precipitation (blue), temperature (red), and solar radiation (orange) to the total difference in GPP_climate-forcing_. A negative (positive) value indicates a lower (higher) mean state of GPP during P2' compared to P1'. All variables are averaged over Eastern China-Mongolia region (40°–52°N and 110°–124°E) as shown in the red box in Fig. 1.

**
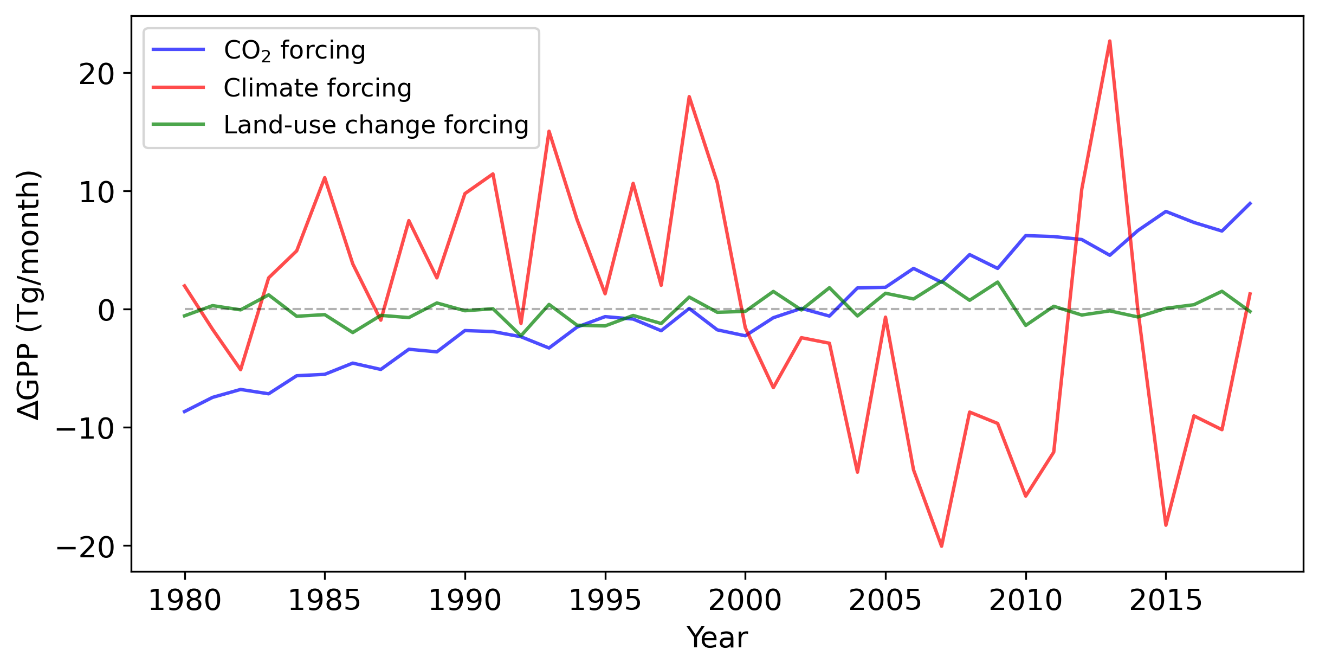
Supplementary Figure. 6** Time series of MME mean of JJA GPP_TRENDY_ anomaly (1980−2018). CO_2_, climate, and land-use change-driven GPP are calculated through the differences between S1 and S0, S2 and S1, S3 and S2, respectively. All values are averaged over Eastern China-Mongolia region (40°–52°N and 110°–124°E). (The “S3” simulation is forced by time-varying atmospheric CO_2_ concentrations, climate, and land-use change.)

**
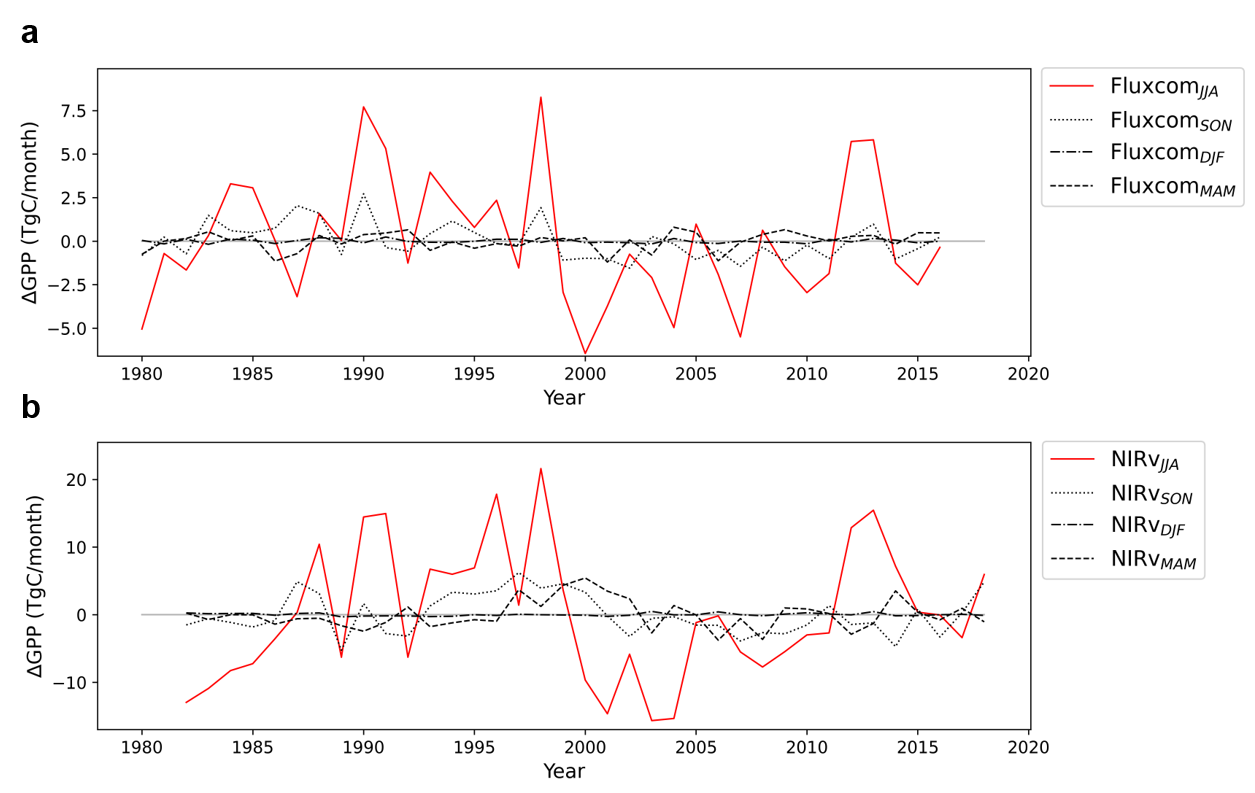
****Supplementary Figure. 7** (a) Time series of seasonal mean GPP_FLUXCOM_ anomaly (1980−2016). (b) Same as (a), but for GPP_NIRv_ anomaly (1982−2018) (June, July, August (JJA); September, October, November (SON); December, January, February (DJF); March, April, May (MAM)). All values are averaged over Eastern China-Mongolia region (40°–52°N and 110°–124°E) as shown in the red box in Fig. 1.
